# Supplementary material for: Chemical Composition, Bioactive Compounds, and Antioxidant Activity of Two Wild Edible Mushrooms Armillaria mellea and Macrolepiota procera from Two Countries (Morocco and Portugal)
Source: Biomolecules. 2021 Apr 14;11(4):575. doi: 10.3390/biom11040575 (PMC8070987; doi:10.3390/biom11040575)
Supplement: Supplementary file 1 [file biomolecules-11-00575-s001.pdf]

biomolecules

MDPI

Supplementary File

Chemical composition, bioactive compounds and antioxidant activity of two wild edible mushrooms *Armillaria mellea* and *Macrolepiota procera* from two countries (Morocco and Portugal)

El Hadi Erbiai [1,2], Luís Pinto da Silva [2] , Rabah Saidi [1], Zouhaire Lamrani [1], Joaquim C.G. Esteves da Silva [2], Abdelfettah Maouni [1,*]

[1] Biology, Environment, and Sustainable Development Laboratory, Higher School of Teachers (ENS), Abdelmalek Essaâdi University, Tetouan, Morocco; erbiai0elhadi@gmail.com (E.H.E.); zahootimaa@gmail.com (F.E.E); r.saidi@uae.ac.ma (R.S.); zh.amrani@yahoo.fr (Z.L), amaouni@uae.ac.ma (A.M.)
[2] Research Centre in Chemistry (CIQUP), DGAOT, Faculty of Sciences, University of Porto, Portugal; luis.silva@fc.up.pt (L.P.S.); jcsilva@fc.up.pt (J.C.G.E.S.)

SUPPLEMENTAL INFORMATION

Supplemental experimental methodology

1.1. Spectrophotometric Determination of Bioactive Compounds

Total Phenolic Content (TFC) was determined by *Folin-Ciocalteu* assay [1]. One ml of extract methanolic solution (5 mg/ml) was mixed with 5 ml of *Folin–Ciocalteu* reagent (previously diluted with distilled water 1:10, v/v) and 4 ml of sodium carbonate solution (7,5%). The tubes were vortex mixed for 15 s and allowed to stand for 30 min at 40 °C in the dark. Then, the absorbance of the solution was measured at 765 nm against the blank. Gallic acid was used to obtain the standard curve (0-250 μg/ml), and the results were mean values ± standard deviations (SD) and expressed as milligrams of gallic acid equivalents (GAE) per gram of dried methanolic extract (dme).

Total Flavonoid Content (TFC) in mushroom samples was carried out according to [2]. Half ml of extract methanolic solution (5 mg/ml) was mixed with 2 ml of distilled water and 0,15 ml of 5% Sodium nitrite solution. After 6 min, 0,15 ml of Aluminium chloride solution (10%) was added and allowed to stand further 6 min. Two ml of Sodium hydroxide solution (4%) was added to the mixture, followed by distilled water until a final volume of 5 ml. The mixture was properly mixed and allowed to stand for 15 min. The intensity of pink color was measured at 510 nm using UV-Vis spectrophotometer against the blank, which contained all reagents except extract samples. (+)-Catechin was used to calculated the standard curve (0-200 μg/ml) and the results were expressed as mg of (+)-catechin equivalents (CE) per gram of dme.

Total Ascorbic Acid Content was determined according to the method used by [1]. A Fine dried mushroom powder (150 mg) was extracted with 10 ml of meta-phosphoric acid (1%), at room temperature with constant

agitation. After 45 min, 100 µl of filtrate (filtration through Whatman paper N° 4) was mixed with 900 µl of 2,6-dichloroindophenol sodium salt solution (3 mg of 2,6-DCPIP and 2,5 mg of sodium bicarbonate dissolved in 100 ml of distilled water). After 30 min, the absorbance was measured at 515 nm against a blank. The ascorbic content was calculated based on the calibration curve of the L-ascorbic acid reference standard (2-120 µg/ml), and the results were expressed as mg of L-ascorbic acid equivalents per gram of dw.

Tannin Content of the sample powder was assayed by the Vanillin-HCL method [3,4]. One g of dried weight was extracted with methanol (50 ml at 28 °C, 24h) and centrifuged for 10 min at 3000 x g then 1 ml from the supernatant was treated with vanillin hydrochloride reagent mixture (1% vanillin in methanol and 8% concentrated HCL in methanol, 1:1 5 ml). After 20 min of incubation, the color developed was measured at 500 nm against the blank. The tannin content was expressed as mg of (+)-catechin equivalents per gram (CEs/g) of dme.

β-Carotene and Lycopene Content were determined following a method previously described by the authors [5,6]. 100 mg of dried methanol extract was vigorously shaken with 10 ml of acetone-hexane (4:6) for 1 min and filtered through Whatman N° 4 filter paper. The filtrate solution absorbance (A) was measured at 453, 505, 645, and 663 nm using a UV-Vis spectrophotometer. Contents of β-carotene and lycopene were calculated according to the following equations: *Lycopene (mg/100 ml)* = $[(0.0458\ A_{663}) + (0.372\ A_{505}) - (0.0806\ A_{453})]$; *β-Carotene (mg/100 ml)* = $[(0.216\ A_{663}) - (0.304\ A_{505}) + (0.452\ A_{453})]$.

1.2. Evaluation of Antioxidant Activity

The antioxidant activity of methanolic extract of mushroom studies was evaluated by three different assays including DPPH radical-scavenging, reducing power, and β-carotene bleaching inhibition assay, according to a slightly modified method previously described by [7]. Trolox was used as a reference standard.

DPPH radical-scavenging activity (RSA) of the samples was measured using the stable free radical DPPH (1.1-diphenyl-2-picrylhydrazyl). A volume of 0,3 ml of methanolic extract solution at different concentrations (8, 4, 2, 1, 0.5, and 0.25 mg/ml) was placed in different test tubes, and 2,7 ml of DPPH radical's solution (6×10^{-5} mol/L) was added to each test tube, then the mixture was shaken vigorously and left to stand at room temperature for 30 min in a dark place to complete the reaction. The absorbance was measured at 517 nm using UV-Vis spectrophotometer against a blank. The RSA was calculated as a percentage of DPPH discoloration using the equation: RSA (%) = $[(A_{DPPH} - A_{Sample})/A_{DPPH}] \times 100$, where A_{DPPH} is the absorbance of the DPPH solution and A_{Sample} is the absorbance of the test extract. The extract concentration providing 50% of RSA (IC_{50}) was calculated from the graph of RSA percentage against extract concentration.

β-Carotene-linoleate bleaching assay. The antioxidant activity of the methanolic extracts was carried out using the β-carotene linoleate model system. Two ml of β-carotene solution (0,2 mg/ml in chloroform) was mixed with 40 mg of linoleic acid and 400 mg of Tween 40, then, the chloroform was removed at 40°C under a vacuum evaporator. The residue obtained

was immediately diluted to 100 ml of distilled water and vigorously shaken to form an emulsion. A 4,8 ml aliquot of the emulsion was transferred into test tubes containing 0,2 ml of different concentrations of each mushroom extract. The mixture was well stirred and the absorbance was measured immediately at zero-time at 470 nm against the blank containing the emulsion without β-carotene. The tubes were incubated in the water bath at 50°C at 100 rpm and the absorbance was measured for the second time at 120 min. A control containing methanol instead of the extract was realized in parallel. β-carotene bleaching inhibition was calculated using the following formula: (%) = (β-carotene content after 2 h of the assay/initial β-carotene content) x 100. The extract concentration providing 50% antioxidant activity (IC_{50}) was calculated by interpolation from the graph of β-carotene bleaching inhibition percentage against extract concentration.

Reducing power by Ferricyanide/Prussian blue assay**.** The volume of 1.5 ml from the various concentrations of the methanolic extract solution of each mushroom was mixed with 1.5 ml of sodium phosphate buffer (0.2 M, pH 6.6) and 1.5 ml of potassium ferricyanide solution (1%). The reaction mixture was incubated for 20 min at 50°C to complete the reaction. After the incubation, 1.5 ml of trichloroacetic acid solution (10%) was added, and the total mixture was centrifuged at 1000 rpm for 8 min. Subsequently, 1.5 ml of the upper layer solution was mixed with 1.5 ml of distilled water and 0.3 ml of ferric chloride solution (0.1%). Then the absorbance of the solution was measured at 690 nm using a UV-Vis spectrophotometer against a blank containing the same solution mixture without mushroom extract. The extract concentration providing 0.5 of absorbance (IC_{50}) was calculated from the graph of absorbance against extract concentration.

Table S1. Sugar compositions of the derivatized methanolic extract by GC-MS

Compound names	*A. mellea* (area %)		*M. procera* (area %)	
	MA	PT	MA	PT
2-deoxy-D-erythropentitol	0.22	0.12	-	-
3-Deoxy-d-mannitol	-	0.25	-	-
Arabinitol	2.62	2.89	0.26	-
Erythritol	0.34	0.81	-	-
Fucitol	1.09	0.91	-	-
Galactitol	16.74	17.21	-	-
Glycerol	3.98	2.67	5.07	24.43
Mannitol	-		0.33	12.48
Threitol	10.52	18.08	-	-
Glyceric acid	-	0.15	-	0.26
D-Gluconic acid	-	0.4	1.95	2
D-Glucopyranosiduronic acid	0.14	0.09	-	-
Tartaric acid	-	0.2	-	-
Threonic acid	0.74	0.6	-	1.36
N-Acetyl-D-galactosamine	1.88	0.32	-	-
N-Acetyl-D-glucosamine	-	0.33	0.01	0.36
D-Fructose	2.8	-	-	0.08
D-Psicose	2.75	0.1	-	0.19
L-Ribulose	-	0.14	-	-
D-Allose	1.37	0.25	0.72	0.39
D-Galactose	1.04	0.29	38.43	1.17
D-Ribose	-	-	-	0.87
L-Arabinose	0.52	-	-	-
D-Xylose	0.74	0.39	-	-
Lactose	-	1.6	1.74	1.32
Lactulose	1.3	-	-	-
Maltose	0.37	0.66	-	-
Mannobiose	2.64	0.32	4.28	-
Trehalose	3.73	0.13	3.66	5.95
Turanose	7.4	-	2.55	2.55

Table S2. Amino acids of the derivatized methanolic extract by GC-MS

Compound Names	*A. mellea* (area %)		*M. procera* (area %)	
	**MA**	**PT**	**MA**	**PT**
Alanine	1	0.41	0.5	2.76
Aspartic acid	0.29	-	0.79	2.45
Canavanine	0.2	-	-	-
gamma-Aminobutyric acid	0.55	-	-	-
Glutamic acid	0.84	-	0.45	2.36
Glutamine	1.22	-	-	1.55
Glycine	0.25	-	-	0.99
Isoleucine	0.83	-	-	-
Phenylalanine	0.71	0.2	0.39	1.27
Pidolic acid	1.39	-	-	3.8
Proline	0.65	-	-	-
Serine	0.88	0.14	0.18	2.61
Threonine	1.12	0.29	1.26	2.45
Tryptophan	-	-	-	2.82
Valine	0.77	0.3	-	-

Table S3. Fatty acids of the derivatized methanolic extract by GC-MS.

Compound Names	*A. mellea* (area %)		*M. procera* (area %)	
	MA	PT	MA	PT
17-Octadecynoic acid	-	0.44	-	-
3,4-Dihydroxybutanoic acid	0.63	-	-	-
5,8,11-Eicosatriynoic acid	-	0.2	-	-
Butyric acid	-	0.88	-	-
Lauric acid	-	0.13	-	-
Leucic acid	-	0.12	-	-
Lignoceric acid	-	-	0.08	0.04
Linolelaidic acid	-	-	5.44	3.69
Linoleic acid	4.83	-	-	-
Palmitic acid	-	4.22	0.42	1.27
Palmitoleic acid	-	1.6	-	-
Petroselinic acid	-	19.83	-	-
Stearic acid	0.65	2.1	0.43	-
Undecylenic acid	-	0.48	-	-

Table S4. Organic acids of the derivatized methanolic extract by GC-MS

Compound Names	*A. mellea* (area %)		*M. procera* (area %)	
	MA	PT	MA	PT
(Aminomethyl)phosphonic acid	-	1.17	-	-
(R)-3-Hydroxybutyric acid	0.09	0.15	-	-
2-Aminoadipic acid	-	-	-	0.17
2-Hydroxyglutaric acid	0.22	0.32	-	-
3,4-Dihydroxybutanoic acid	-	-	-	0.57
3-Hydroxypyruvic acid	-	-	-	0.44
4-Hydroxybutanoic acid	0.09	0.59	-	-
Citric acid	-	-	0.5	-
Fumaric acid	2.2	-	-	-
Glyceric acid	0.31	-	-	-
Glycolic acid	0.11	0.26	-	-
Glyoxylic acid	-	-	-	0.71
Lactic acid	1.24	2.02	1.12	0.1
Maleic acid	-	-	1.23	3.11
Malic acid	4.65	0.82	2.54	2.34
Succinic acid	2.29	2.45	4.93	-
Urea	-	-	10.68	-

Table S5. Rest of the biomolecule constituents of the derivatized methanolic extract by GC-MS

Compound Names	*A. mellea* (area %)		*M. procera* (area %)	
	MA	PT	MA	PT
1,2,3-Butanetriol	0.1	0.47	-	-
1,5-Pentanediol	-	0.45	-	-
2-(3-Nitrophenyl)ethanol	-	-	-	0.2
2,3-Butanediol	0.34	0.89	-	-
2,4,7-Trimethyl-1,8-naphthyridine	-	-	0.52	1.44
2-Monoolein	0.13	0.2	-	0.37
2-Pentanol	-	0.3	-	-
5-Chloro-1H-indole-2,3-dione	-	-	-	0.15
5-Methyluridine	1.03	-	3.15	0.58
Adenosine	1.41	0.16	0.57	0.97
Brassicasterol acetate	0.1	0.12	-	-
Calcitriol (Vitamin D)	-	-	-	0.13
Chloro(3-chloropropyl)dimethylsilane	2.19	3.78	-	2
Deanol	0.3	-	-	-
Dehydroergosterol	-	-	0.03	0.06
Diethylamine	-	-	-	0.16
Diethylene glycol	0.14	-	-	-
Diphenylphosphine	-	0.86	-	-
Ergosta-7,22-dien-3-ol, (3β,22E)-	-	-	0.16	0.18
Ergosterol	0.75	1.44	0.5	1.1
Glycerol monostearate	0.1	0.32	-	0.22
Glycerol-3-phosphate	1.71	0.08	-	0.79
Glyceryl monooleate	-	1.46	-	-
Glyceryl palmitate	-	0.21	-	0.11
Inosine	-	-	-	1.55
Pipecolic acid	-	-	4.73	-
Silane, dimethyl(2-octyloxy)propoxy-	-	0.97	-	-
Thymine	-	0.23	-	-
Trifluoroacetamide	0.71	-	-	-
Uridine	0.06	-	0.4	0.77

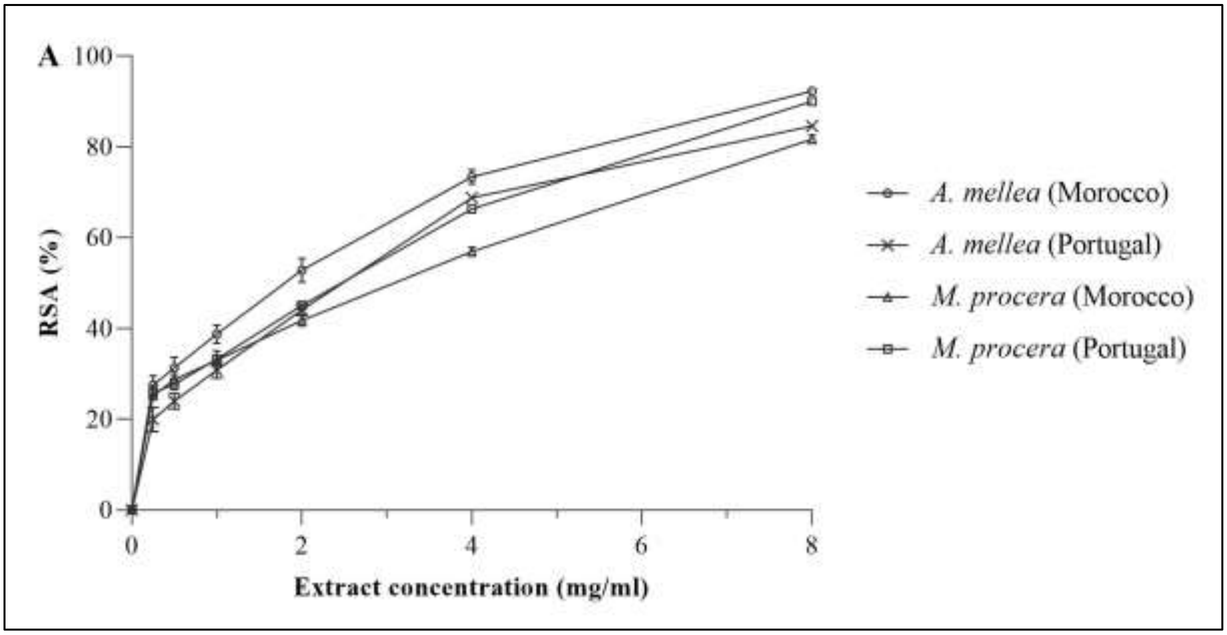

Figure S1. Radical-scavenging activity on DPPH radicals. Each value is expressed as mean ± SD (n = 3).

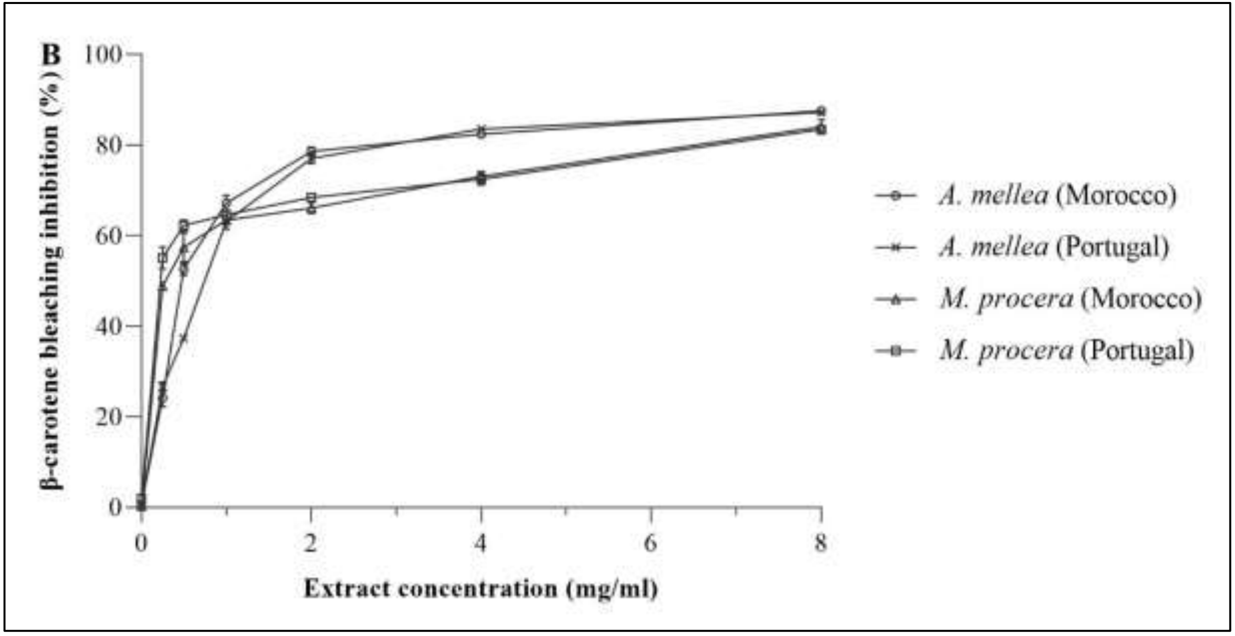

Figure S2. Lipid peroxidation inhibition measured by the β-carotene bleaching inhibition. Each value is expressed as mean ± SD (n = 3).

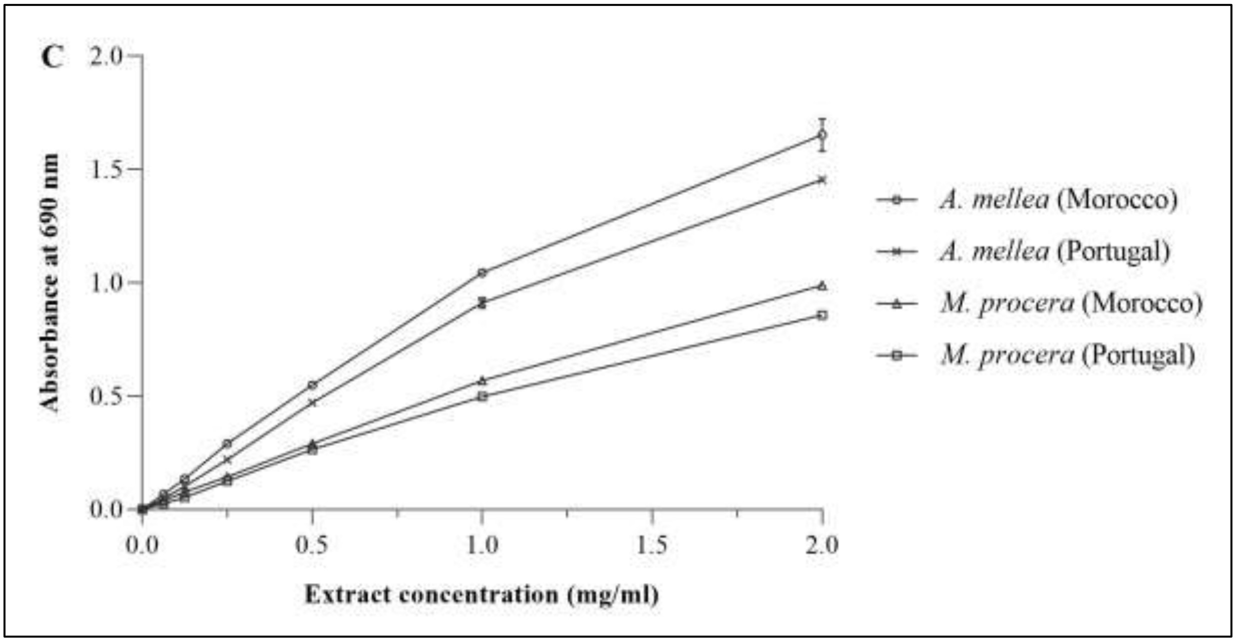

Figure S3. Reducing power. Each value is expressed as mean ± SD (n = 3).

References

1. Heleno, S.A.; Barros, L.; Sousa, M.J.; Martins, A.; Santos-Buelga, C.; Ferreira, I.C.F.R. Targeted Metabolites Analysis in Wild Boletus Species. *LWT - Food Sci. Technol.* **2011**, *44*, 1343–1348, doi:10.1016/j.lwt.2011.01.017.
2. Pereira, E.; Barros, L.; Martins, A.; Ferreira, I.C.F.R. Towards Chemical and Nutritional Inventory of Portuguese Wild Edible Mushrooms in Different Habitats. *Food Chem.* **2012**, *130*, 394–403, doi:10.1016/j.foodchem.2011.07.057.
3. Ghate, S.D.; Sridhar, K.R. Bioactive Potential of Lentinus Squarrosulus and Termitomyces Clypeatus from the Southwestern Region of India. **2017**, 12.
4. Hagerman, A.E. Vanillin Assay. **2002**.
5. Barros, L.; Ferreira, M.-J.; Queirós, B.; Ferreira, I.C.F.R.; Baptista, P. Total Phenols, Ascorbic Acid, β-Carotene and Lycopene in Portuguese Wild Edible Mushrooms and Their Antioxidant Activities. *Food Chem.* **2007**, *103*, 413–419, doi:10.1016/j.foodchem.2006.07.038.
6. Nagata, M.; Yamashita, I. Simple Method for Simultaneous Determination of Chlorophyll and Carotenoids in Tomato Fruit. *NIPPON SHOKUHIN KOGYO GAKKAISHI* **1992**, *39*, 925–928, doi:10.3136/nskkk1962.39.925.
7. Heleno, S.A.; Barros, L.; Sousa, M.J.; Martins, A.; Ferreira, I.C.F.R. Tocopherols Composition of Portuguese Wild Mushrooms with Antioxidant Capacity. *Food Chem.* **2010**, *119*, 1443–1450, doi:10.1016/j.foodchem.2009.09.025.
